# The Carnitine Palmitoyltransferase 1A Inhibitor Teglicar Shows Promising Antitumour Activity against Canine Mammary Cancer Cells by Inducing Apoptosis

**DOI:** 10.3390/ph16070987

**Published:** 2023-07-10

**Authors:** Nunzio Antonio Cacciola, Fabrizia Sepe, Salvatore Fioriniello, Orsolina Petillo, Sabrina Margarucci, Marcello Scivicco, Gianfranco Peluso, Anna Balestrieri, Giovanna Bifulco, Brunella Restucci, Lorella Severino

**Affiliations:** 1 Department of Veterinary Medicine and Animal Production, University of Naples Federico II, Via F. Delpino 1, 80137 Naples, Italy; nunzioantonio.cacciola@unina.it (N.A.C.); marcello.scivicco15@gmail.com (M.S.); giovanna.bifulco@unina.it (G.B.); lseverin@unina.it (L.S.); 2 Research Institute on Terrestrial Ecosystems (IRET), UOS Naples-National Research Council (CNR), Via Pietro Castellino 111, 80131 Naples, Italy; orsolina.petillo@cnr.it (O.P.); sabrina.margarucci@cnr.it (S.M.); gianfranco.peluso@unicamillus.org (G.P.); 3 Institute of Experimental Endocrinology and Oncology “G. Salvatore” (IEOS), National Research Council (CNR), Via Pietro Castellino 111, 80131 Naples, Italy; fabriziasepe93@gmail.com; 4 Institute of Genetics and Biophysics “A. Buzzati-Traverso“ (IGB), National Research Council (CNR), Via Pietro Castellino 111, 80131 Naples, Italy; salvatore.fioriniello@igb.cnr.it; 5 Faculty of Medicine and Surgery, Saint Camillus International University of Health and Medical Sciences, Via di Sant’Alessandro 8, 00131 Rome, Italy; 6 Food Safety Department, Istituto Zooprofilattico Sperimentale del Mezzogiorno, Via Salute 2, 80055 Portici, Italy; anna.balestrieri@izsmportici.it

**Keywords:** canine mammary tumours, apoptosis, teglicar, carnitine palmitoyl transferase 1A, veterinary pharmacology

## Abstract

Canine mammary tumours (CMTs) are the most common cancer in intact female dogs. In addition to surgery, additional targeted and non-targeted therapies may offer survival benefits to these patients. Therefore, exploring new treatments for CMT is a promising area in veterinary oncology. CMT cells have an altered lipid metabolism and use the oxidation of fatty acids for their energy needs. Here we investigated the tumoricidal effects of teglicar, a reversible inhibitor of carnitine palmitoyl transferase 1A (CPT1A), the rate-limiting enzyme for fatty acid import into mitochondria, on two CMT cells, P114 and CMT-U229. Viability and apoptosis were examined in CMT cells using the crystal violet assay, trypan blue assay, and flow cytometry analysis. The expression of mediators of apoptosis signalling (e.g., caspase-9, caspase-8, and caspase-3) was assessed by quantitative real-time polymerase chain reaction and western blot analyses. Teglicar was able to decrease cell viability and induce apoptosis in P114 and CMT-U229 cells. At the molecular level, the effect of teglicar was associated with an upregulation of the mRNA expression levels of *caspase-9*, *caspase-8,* and *caspase-3* and an increase in their protein levels. In summary, our results show that teglicar has a potential effect against CMTs through the induction of apoptotic cell death, making it a promising therapeutic agent against CMTs.

## 1. Introduction

Over the last twenty years, the development of therapies for breast cancer (HBC) has incredibly increased the survival rate and quality of life of carcinoma patients [1]. However, there are still many challenges to treating this pathology, such as resistance to chemotherapeutic agents [2]. In recent years, there has been a focus on promoting comparative studies on the prognosis and treatment of breast cancer. Numerous authors have confirmed canine mammary tumours (CMTs) as a good spontaneous model for the study of HBC, as they share many similar histological, genetic, morphological, molecular, clinical, and environmental aspects [3]. CMTs represent the majority of tumours in female dogs, with an incidence of 50–70% [4]. Currently, the most reliable treatment for CMT is the surgical removal of the tumour and its local lymph nodes. Therefore, following the One Health concept, despite the extensive research on HBC, new and improved therapeutic protocols for CMT are needed [4]. Alteration of energy metabolism is considered a typical feature of cancer [5]. In addition to dysregulation of glucose metabolism, lipids also play an important role in cancer progression since they can be degraded by fatty acid β-oxidation (FAO) to provide energy once taken up into cells [6]. It has been reported that FAO promotes tumour growth by enhancing ATP production under metabolic stress conditions [7]. The carnitine system (CS) appears to play an important role between lipid catabolism and cell cycle regulation, as shown by several studies in many types of neoplasms [8,9]. Carnitine palmitoyltransferase (CPT) is an enzyme that exists in two isoforms, CPT1 and CPT2, which are mainly found in mitochondria. CPT1A, which is localised at the outer mitochondrial membrane, has the task of catalysing the conversion of acyl-CoA to acylcarnitine. The next step involves the transport of long-chain fatty acids (e.g., palmitate) into the intermembrane space and the subsequent conversion of acylcarnitine back to CoA for β-oxidation, which is catalysed by CPT2, localised to the inner membrane [8,9,10]. Several studies show that high CPT1 levels are closely associated with tumour progression in breast cancer, making it an important target for pharmacotherapy [11,12,13,14]. Indeed, these studies suggest that inhibition of CPT1A reduces proliferation rates, decreases cancer cell chemoresistance, and increases apoptosis. Inhibition of CPT1A is usually achieved by etomoxir treatment, although there is evidence that this inhibitor can be toxic at high concentrations and have off-target effects [15,16]. For this reason, the reversible and selective CPT1A inhibitor, teglicar [(R)-N-(tetradecylcarbamoyl)-aminocarnitine, also known as ST1326], has been developed. Many studies show that teglicar reduces the proliferation of lymphoma and leukaemia cells [14,17,18]. To date, not much is known about CS in CMTs. Thus, our recent study investigated the expression and downregulation of CPT1A in CMT tissues and cells [19]. Based on what has been said so far, in this work we decided to investigate the effect of teglicar on CMT cells and noncancerous canine epithelial cells. We mainly focused on the CMT cell lines P114 and CMT-U229 to evaluate the teglicar antitumor properties by studying its effects on the caspase signalling pathway.

## 2. Results

### 2.1. Teglicar Inhibits the Viability of Canine Mammary Tumour Cells

**Figure 1:**
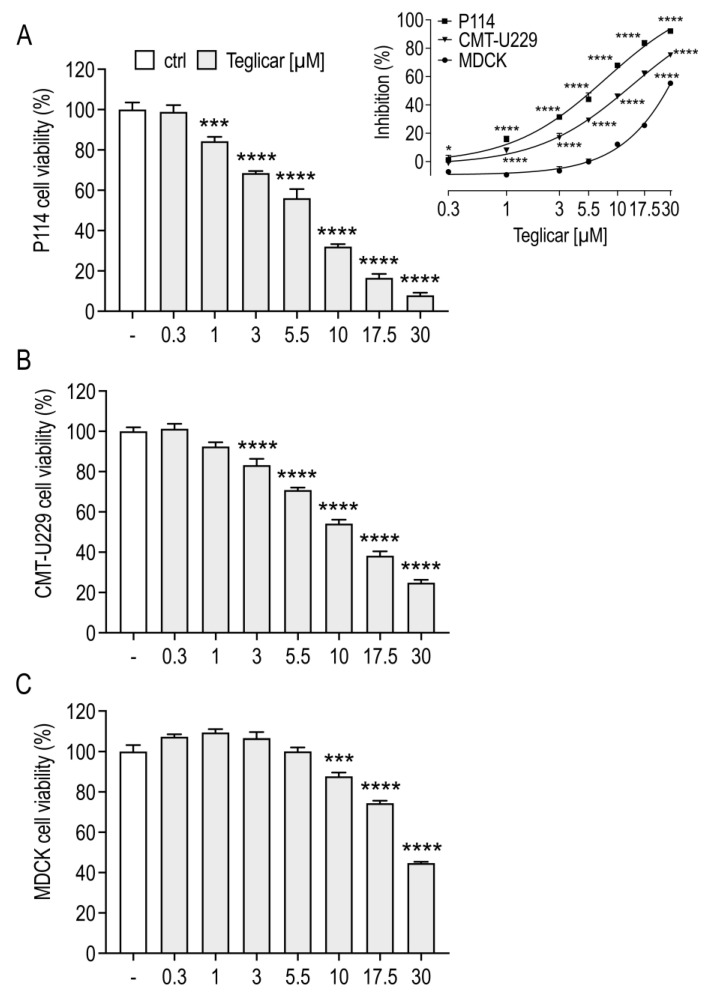
Teglicar selectively inhibits the viability of mammary tumour cells. Cell viability was analysed by crystal violet assay in (**A**) canine P114 and (**B**) canine CMT-U229 mammary tumour cells and (**C**) canine MDCK noncancerous epithelial cells. Cell viability was determined after 24 h exposure to different concentrations of Teglicar (0.3–30 µM). The data represent the mean ± SEM of three different experiments. *** *p* < 0.001; **** *p* < 0.0001 vs. ctrl (untreated cells). The insert shows the dose-response curves generated using the function “Non-linear curve fit—log (inhibitor) vs. response” with the software GraphPad PRISM v9.0. * *p* < 0.05; **** *p* < 0.0001.

To test whether teglicar exerts a cytotoxic effect, we investigated its tumour-killing effect on P114 and CMT-U229 cells using the crystal violet assay. As shown in Figure 1A, teglicar at concentrations of 1, 3, 5.5, 10, 17.5, and 30 µM significantly inhibited P114 cell viability by 16, 32, 44, 68, 84, and 92%, respectively. The same trend was observed when assessing the cytotoxic effect of teglicar on CMT-U229 cells. At concentrations of 3, 5.5, 10, 17.5, and 30 µM, the viability of CMT-U229 cells significantly decreased by 17, 30, 46, 62, and 75%, respectively (Figure 1B). The effect of teglicar was also tested on noncancerous canine epithelial cells (MDCK). As shown in Figure 1C, at concentrations of 0.3 to 5.5 µM, teglicar had no effect on MDCK cell viability, whereas at concentrations of 10 and 17.5 µM, MDCK cell viability was slightly impaired (12% at 10 µM and 26% at 17.5 µM). At the highest concentration tested (30 µM), MDCK cell viability was inhibited by 55%. Based on the IC50 results for the analysed cell lines (Table 1), the P114 and CMT-U229 cells appear to be more sensitive to teglicar treatment compared to the MDCK cells (Figure 1, insert), suggesting a selective cytotoxic effect against CMT cells.

### 2.2. Teglicar Indues Cell Death in Canine Mammary Tumour Cells

**Figure 2:**
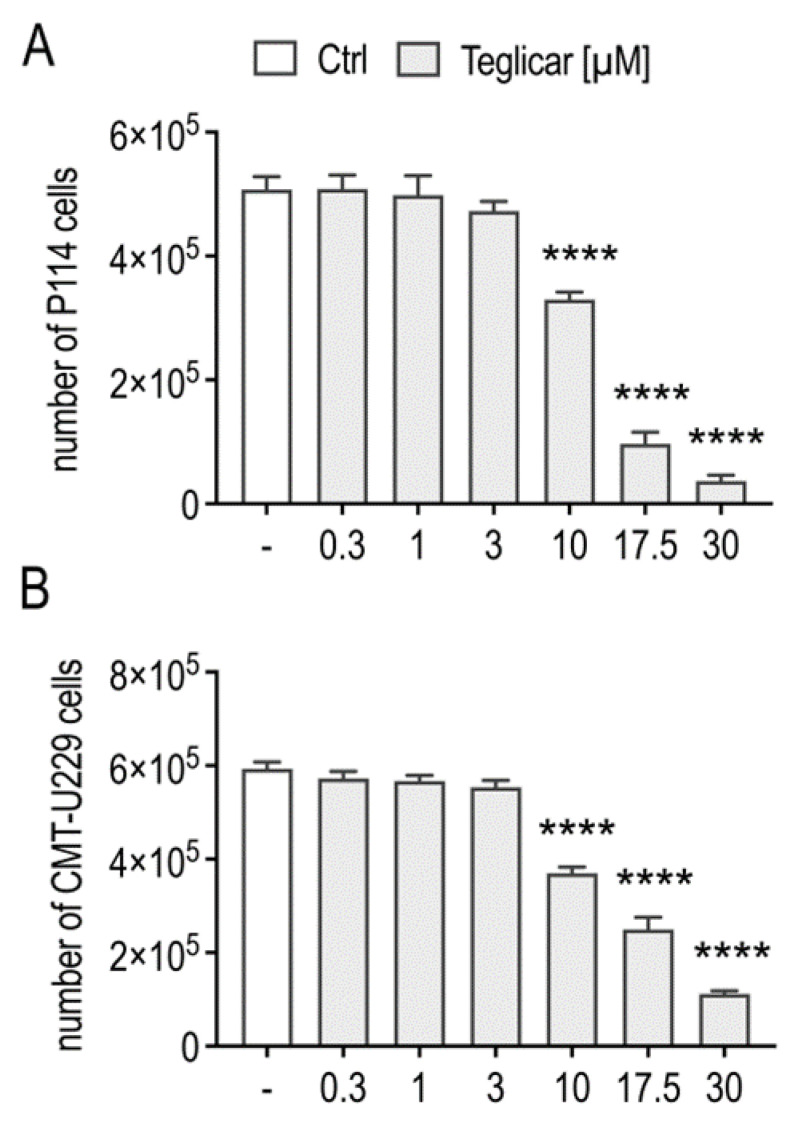
Teglicar induces growth arrest in mammary tumour cells. Cell viability was analysed by trypan blue assay in (**A**) P114 cells and (**B**) CMT-U229 cells. Cell viability was determined after 24 h exposure to different concentrations of Teglicar (0.3–30 µM). The data represent the mean ± SEM of three different experiments. **** *p* < 0.0001 vs. ctrl (untreated cells).

To distinguish between cell death and possible growth arrest by teglicar, the viability of P114 and CMT-U229 cells in response to teglicar treatment was monitored by counting viable cells using the trypan blue exclusion procedure, as described in the Section 4. We found that teglicar treatment also resulted in a significant concentration-dependent decrease in the number of viable cells of P114 and CMT-U229, indicating cell death when compared to the number of cells treated with vehicle (Figure 2A,B).

### 2.3. Teglicar Induces Morphological Changes in CMT Cells

**Figure 3:**
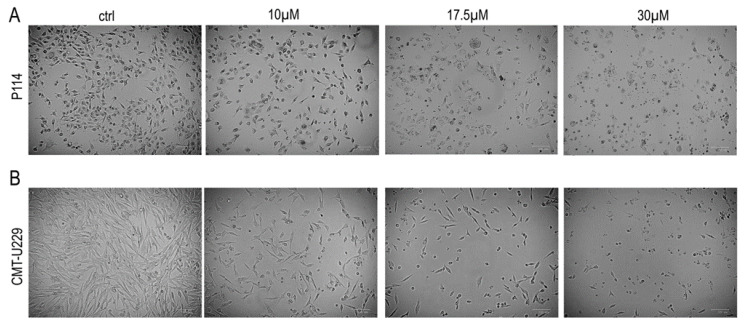
Teglicar induces morphological changes in canine mammary tumour cells. (**A**) P114 and (**B**) CMT-U229 cells were incubated with vehicle alone (control, ctrl) or with 10, 17.5, or 30 μM Teglicar for 24 h, and the morphological changes of the cells were observed using the ZOE™ Fluorescent Cell Imager (Biorad, Milan, Italy) (magnification 100×).

We examined the morphology of P114 and CMT-U229 cells treated with teglicar or vehicle using phase contrast microscopy. As shown in Figure 3A, examination of vehicle-treated P114 cells showed that these cells had normal morphological characteristics, spread regularly, and adhered firmly to the bottom of the Petri dish. These cells maintained these characteristics until the treatment step was completed. In contrast, P114 cells treated with 10 µM teglicar exhibited a long, thin, spindle-like shape. A marked reduction in cell volume and shrinkage of the cells (indicating cell death) were observed when the P114 cells were treated with 17.5 µM teglicar. When exposed to 30 μM teglicar, most P114 cells detached from the culture medium and floated in it. These cells showed distinct morphological abnormalities. They lost their typical morphology and had an irregular shape, appearing smaller and rounder. Similar results were obtained when CMT-U229 cells were subjected to the same type of treatment (Figure 3B).

### 2.4. Teglicar Triggers Apoptosis in CMT Cells

**Figure 4:**
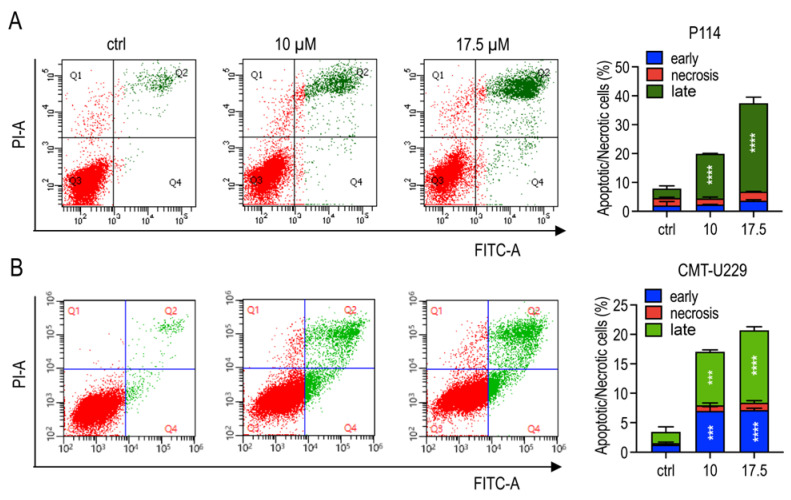
Teglicar induces apoptosis in CMT cells. (**A**,**B**) Representative dot plots of P114 and CMT-U229 cells treated with 10 or 17.5 µM teglicar for 24 h, analysed by flow cytometry after double staining of the cells with annexin-V FITC and PI. Q1: Annexin-VFITC-/PI+ (upper left quadrant/necrotic cells); Q2: Annexin-V-FITC+/PI+ (upper right quadrant/late apoptotic cells); Q3: Annexin-V-FITC-/PI- lower left quadrant/viable cells); and Q4: Annexin-V-FITC+/PI- (lower right quadrant/early apoptotic cells). Bars show the percentage of apoptotic and necrotic cell populations in P114 and CMT-U229 cells after 24 h incubation with vehicle alone (control, ctrl) or with teglicar (10 or 17.5 µM). Data are presented as mean ± SEM of two independent experiments. *** *p* < 0.001; **** *p* < 0.0001 vs. ctrl (untreated cells).

The ability of teglicar to inhibit cell viability, as well as the clear observation of morphological changes often associated with apoptosis, prompted us to investigate whether inhibition of cell viability actually induces apoptosis in P114 and CMT-U229 cells. Therefore, the inhibitory effect of teglicar on the viability of anaplastic and highly aggressive P114 cells was investigated by flow cytometry analysis. As shown in Figure 4A, when P114 cells were treated with 10 or 17.5 µM Teglicar for 24 h, there was a significant increase in the percentage of late apoptotic cells (Annexin V+/PI + staining), which increased from ~16% at 10 µM to 30.7% at 17.5 µM. The effect of teglicar on apoptosis induction was also investigated in CMT-U229 cells. As shown in Figure 4B, it was observed that the percentage of early apoptosis (region Q4) was increased in CMT-U229 cells treated with 10- or 17.5-µM Teglicar compared to treatment with vehicle (6.99% vs. 1.28% at 10 µM; 7.13% vs. 1.28% at 17.5 µM). A significant increase in late-stage apoptotic/necrotic cells (Annexin V+/PI+) was also observed at 10 µM (9.1% vs. 1.92%) and 17.5 µM Teglicar (12.31% vs. 1.92%), suggesting that Teglicar was able to induce apoptotic cell death in CMT-U229 cells as well.

### 2.5. Teglicar-Induced Apoptosis Is Mediated by Caspases Activation

**Figure 5:**
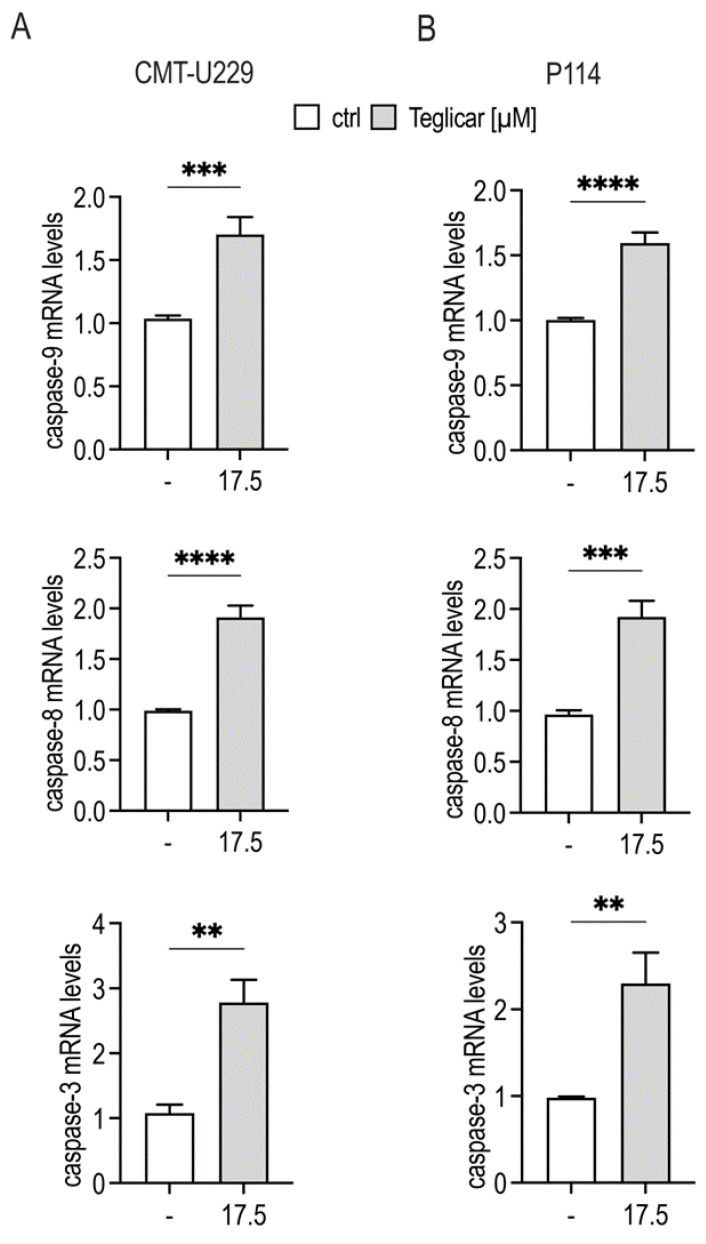
Teglicar treatment increases caspase gene expression in canine mammary tumour cells. CMT-U229 and P114 cells were treated with 17.5 µM teglicar for 24 h. The expression of mRNAs was analysed by quantitative real-time PCR and normalised with *RPS5* expression. (**A**) Relative mRNA expression of *caspase-9*, *caspase-8,* and *caspase-3* in CMT-U229 cells. (**B**) relative mRNA expression of *caspase-9*, *caspase-8,* and *caspase-3* in P114 cells. Data are presented as mean ± SEM of three independent experiments. ** *p* < 0.01; *** *p* < 0.001; **** *p* < 0.0001 vs. ctrl (untreated cells).

To determine a possible mechanism responsible for the inhibition of cell viability induced by teglicar, we investigated its effects on genes involved in apoptotic signalling in both P114 and CMT-U229 cells. In these experiments, the submaximal concentration of teglicar (17.5 µM) was used to allow the detection of enhanced effects by apoptosis induction. Using quantitative real-time PCR, it was found that the mRNA expression levels of *caspase-9*, *caspase-8,* and *caspase-3* were increased in the treated P114 and CMT-U229 cells compared to the vehicle-treated cells (Figure 5A,B).

**Figure 6:**
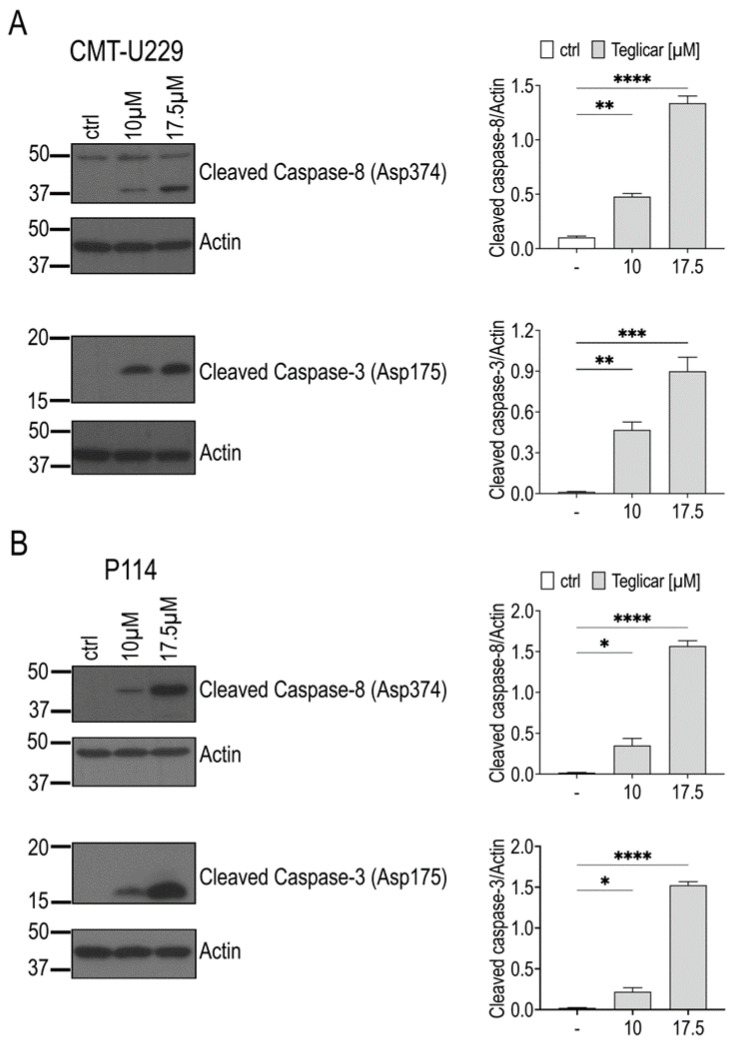
Teglicar treatment increases caspase protein expression in canine mammary tumour cells. (**A**) CMT-U229 and (**B**) P114 cells were treated with or without the indicated concentrations of teglicar for 24 h, and protein levels of cleaved caspase-8 and cleaved caspase-3 were assayed by western blotting. The membrane of cleaved caspase-8 was stripped and reprobed with an anti-actin antibody. Data are presented as mean ± SEM of three independent experiments. * *p* < 0.05; ** *p* < 0.01; *** *p* < 0.001; **** *p* < 0.0001. Figures S1 and S2: Original blots for CMT-U229 and P114 cells of Figure 6A,B.

To verify whether the changes in caspases observed at the mRNA level also occurred at the protein level after treatment with teglicar, caspase-8 and caspase-3 protein levels were determined by Western blot analysis. As shown in Figure 6A,B, a significant increase in the cleavage of caspases 8 and 3 was observed after 24 h in CMT-U229 and P114 cells treated with 10 or 17.5 μM teglicar compared to the cells treated with vehicle (ctrl).

## 3. Discussion

In the present study, we reported the anticancer effect of teglicar in canine mammary tumour (CMT) cells. We demonstrated that teglicar suppresses CMT cell viability and leads to programmed cell death of CMT cells by activating crucial regulators of the apoptotic pathway, suggesting that treatment with teglicar may represent a possible novel therapeutic strategy for CMT. According to the literature, cancer is one of the most common causes of death in companion animals, and of all neoplasms, mammary tumours are the second most common cancer in female dogs [20,21]. Chemotherapy is considered an important adjuvant therapy after surgical treatment of malignant mammary tumours in dogs [4]. It helps to improve overall survival and prevent tumour recurrence [22]. Nevertheless, several conventional anticancer drugs may produce certain effects at the beginning of treatment but may develop resistance and lose efficacy with continued treatment [23]. The mechanisms that would accurately explain acquired resistance to chemotherapy are not known for most animal tumours. Nevertheless, there are numerous findings on the mechanisms of drug resistance in humans, and there are some reports of molecular alterations in resistant tumours in dogs [24]. Chemotherapy research in veterinary oncology is making steady progress, but due to the lack of effective chemotherapeutic agents, dogs with malignant tumours currently have high recurrence rates [25] and a poor prognosis [26]. Despite this, chemotherapy is often used in dogs with tumours that are at high risk of metastasis and/or recurrence [20]. From an application perspective, the development of new chemotherapeutic agents for the treatment of mammary tumours in dogs is therefore urgently needed. Several studies have identified carnitine palmitoyl transferase 1 A (CPT1A) as a druggable target for various cancers, and targeting CPT1A has been shown to have anti-cancer effects [27,28,29,30,31]. The aminocarnitine derivative teglicar (R)-N-(tetradecylcarbamoyl)-aminocarnitine (ST1326), a selective and reversible inhibitor of CPT1A originally used as a drug candidate for diabetes therapy [32,33], also plays a central role in the therapy of certain cancers. Previous studies have shown that teglicar exerts antitumor activity and is able to reduce the viability and growth of lymphoma, leukaemia, chronic lymphocytic leukaemia, and cervical cancer in preclinical studies [14,17,18,34]. Consistent with these findings, in the present study, using two different methods to determine cell viability, we found that teglicar significantly inhibited the viability and growth of canine mammary cancer cells CMT-U229 and P114. Interestingly, when we investigated the cytotoxic effect of teglicar on canine noncancerous epithelial cells (MDCK), we found that this compound exhibited a very low cytotoxic effect, with cell viability of 88% at 10 µM and 74% at 17.5 µM, indicating a selective cytotoxic effect against cancer cells and thus a promising safety profile. The onset of apoptosis is generally characterised by several morphological features and energy-dependent biochemical mechanisms [35]. Based on our findings in this report, morphological changes such as cell shrinkage, membrane blebbing, and the presence of apoptotic bodies were observed in teglicar-treated CMT-U229 and P114 cells using light microscopy, indicating an apoptotic phenotype. Noteworthy, the morphological changes were dependent on the concentration of the drug used, with confirmed and observable phenotypic changes occurring after 24 h of treatment. It has been previously reported that teglicar is able to increase the number of apoptotic cells in both human leukaemia and cervical cancer cells in a concentration-dependent manner [18,34]. Therefore, to gain a better insight into the mode of cell death induced by teglicar in CMT cells, we performed a flow cytometry analysis. Our results showed that the percentage of apoptotic cells in the teglicar-treated cells increased in a concentration-dependent manner compared to the vehicle-treated cells. Caspases are cysteine proteases that specifically cleave their substrate proteins behind an aspartate residue [36]. In order for them to develop their full enzymatic activity, they must be cleaved at specific internal aspartate residues that separate large and small subunits [37,38]. Several studies have shown that caspases are activated during apoptosis in a self-reinforcing cascade. Mechanistically, using quantitative-real-time transcription polymerase chain reaction and Western blot analyses, the results of this study demonstrate that teglicar-induced apoptosis appears to be due, at least in part, to the increase in mRNA expression levels of *Caspase-8*, *Caspase-9*, and *Caspase-3*, as well as increased protein expression of cleaved caspase-8 and cleaved caspase-3. These findings are consistent with a previous study in which the authors demonstrated that teglicar induces apoptosis and inhibits the growth of human cervical cancer cells via a caspase-dependent signalling pathway [34]. In addition, our results are also consistent with a previous report [28]**,** in which the authors demonstrated that inhibition of CPT1A led to programmed cell death through upregulation of pro-apoptotic genes, including *Caspase-9*.

In closing, the present study demonstrated that teglicar inhibits the viability of the P114 and CMT-U229 canine mammary cancer cell lines and induces apoptosis. The induction of apoptosis can be achieved by upregulating the mRNA expression levels of *Caspase-8*, *Caspase-9,* and *Caspase-3* and by cleaving the caspase-8 and caspase-3 proteins. In summary, our results provide some experimental evidence for the possible therapeutic effect of teglicar on CMT cells.

## 4. Materials and Methods

### 4.1. Reagents and Antibodies

Teglicar (purity ≥ 99%, Cat. No. 870853), crystal violet powder (Cat. No. C0775), cOmplete™, EDTA-free Protease Inhibitor Cocktail (Cat. No. 11836170001), PhosSTOP™ (Cat. No. 4906837001), Bovine Serum Albumin (Cat. No. A7030), Immobilon Crescendo Western HRP substrate (Cat. No. WBLUR0500), and X-ray film (Fuji Film, Tokyo, Japan), and trypan blue (Cat. No. T8154) were purchased from Merk Life Science S.r.l. (Milan, Italy). Annexin V-FITC Apoptosis Detection Kit (Cat. No. AD10) was purchased from Dojindo Molecular Technologies Inc. (Munich, Germany). The RDP Trio^TM^ reagent (Cat. No. MB566) was purchased from HiMedia Laboratories (Maharashtra, India). QuantiTect Reverse Transcription Kit (Cat. No. 205313) was purchased from Qiagen (Hilden, Germany), and SsoAdvanced Universal SYBRGreen Supermix (Cat. No. 1725271), DC™ Protein Assay Kit II (Cat. No. 5000111), 4× Laemmli Sample Buffer (Cat. No. 1610747), Nitrocellulose Membrane 0.2 µm (Cat. No. 1620112), and Non-Fat Dry Milk (Cat. No. 10004504) were purchased from Bio-Rad Laboratories (Milan, Italy). Radioimmunoprecipitation assay buffer (RIPA buffer, Cat. No. 9806), Cleaved caspase-3 (Cat. No. 9664S), cleaved caspase-9 (Cat. No. 20750S), cleaved caspase-8 (Cat. No. 9496S), goat anti-rabbit IgG (Cat. No. 7074S), and anti-mouse IgG (Cat. No. 7076S) were purchased from Cell Signalling Technology Inc. (Danvers, MA, USA). β-Actin (Cat. No. SC -47778) was acquired from Santa Cruz Biotechnology Inc. (Dallas, MA, USA).

### 4.2. Cell Culture

CMT-U229 cells (an atypical benign mixed tumour cell line) were kindly provided by Prof. Dr. Eva Hellmen from Uppsala University, while P114, a canine anaplastic mammary carcinoma cell line, was kindly provided by Prof. Dr. Gerard Rutteman (Department of Clinic Science and Companion Animals, University of Utrecht, The Netherlands). MDCK cells were kindly provided by Prof. Dr. Serena Montagnaro (Department of Veterinary Medicine and Animal Production, University of Naples Federico II, Italy). CMT-U229 cells were grown in Roswell Park Memorial Institute (RPMI) medium (Gibco, NY, United States), while P114 cells were grown in Dulbecco’s Modified Eagle’s Nutrient Mixture F-12 (DMEM/F12) medium (Gibco, NY, United States). MDCK cells were grown in Dulbecco’s Modified Eagle Medium (DMEM) medium (Gibco, NY, USA). All the cells were supplemented with 10% foetal bovine serum (FBS, Gibco), 100 U/mL penicillin, and 100 μg/mL streptomycin in a controlled humidified cell culture incubator (37 °C, 5% CO_2,_ and 95% humidity). The cell lines were routinely tested for mycoplasma contamination.

### 4.3. Crystal Violet Assay

CMT-U229, P114, and MDCK cells were seeded at a density of 3 × 10^3^, 4 × 10^3^, and 1 × 10^4^ cells/well, respectively, in a 96-well plate and kept at 5% CO_2_ and 37 °C for 24 h to allow them to settle to the bottom overnight. Then the medium was replaced with a fresh complete medium containing 1% FBS and increasing concentrations (0–30 μM) of teglicar, and the cells were incubated for 24 h. Then 50 μL of 0.5% crystal violet staining solution was added to each well, and the plate was incubated for 20 min at room temperature (RT) on an orbital shaker. The plate was washed four times with tap water and dried overnight at RT. Then 200 μL of methanol was added to each well, and the cells were incubated at RT for 20 min. The optical densities (OD) of the wells were measured at 570 nm using a microplate reader (BioTek™ Cytation™ 3, Winooski, VT, USA). All samples were analysed in triplicate, and the mean value for each experiment was calculated. The effect of teglicar on viability was estimated as a percentage of cell viability; cells treated with a vehicle were considered 100% viable.

### 4.4. Trypan Blue Assay

CMT-U229 and P114 cells were seeded at a density of 0.75 × 10^5^ cells/well and 1.2 × 10^5^ cells/well, respectively, in a 6-well plate and maintained in 5% CO_2_ at 37 °C for 24 h to allow them to settle to the bottom overnight. The medium was then replaced with a fresh complete medium containing 1% FBS and increasing concentrations (0–30 μM) of teglicar. After 24 h incubation, cells were trypsinised and centrifuged (300× *g* for 4 min). The pellet was suspended in a culture medium, and 10 μL of the cell suspension was mixed with an equal volume of a 0.4% trypan blue solution. Live (colourless) and dead (blue-stained) cells were counted and viability was determined using the Bio-Rad TC20™ Automated Cell Counter (Bio-Rad).

### 4.5. Annexin V-FITC Apoptosis Detection

Apoptosis was assayed using the Annexin V-FITC Apoptosis Detection Kit according to the manufacturer’s instructions. Briefly, P114 or CMT-U229 cells were treated with or without teglicar for 24 h. Detached and adherent cells were collected and incubated with Annexin V and PI for 15 min at room temperature in the dark. Events for live, early, necrotic, and late apoptotic cells were analysed using the BD FACSCanto II system and elaborated using the DIVA software (BD Biosciences, Milan, Italy). For each condition, at least 20,000 events were recorded.

### 4.6. RNA Isolation, cDNA Synthesis, and Quantitative Real-Time PCR (qRT-PCR)

After treating the cells with or without teglicar for 24 h, the cells were harvested, washed with ice-cold PBS, and total RNA was isolated using the RDP Trio^TM^ reagent according to the manufacturer’s protocol. Total RNA (1 µg) from each cell line was reverse transcribed using the QuantiTect Reverse Transcription Kit (Qiagen, Germany). The final reaction mixtures were incubated at 95 °C for 3 min, 42 °C for 30 min, and 4 °C overnight. The cDNAs were used as templates for qRT-PCR using Power SYBR Green PCR Master Mix (Bio-Rad), following the manufacturer’s instructions. The primer sequences for canine *Rps -5*, *Caspase-3*, *Caspase-8,* and *Caspase-9* are listed in Table 2. Relative mRNA expression levels were calculated using the 2^−ΔΔCt^ method. The transcription level of the canine genes *Caspase-3*, *Caspase-8,* and *Caspase-9* was normalised to *Rps-5* and determined in two replicates by qRT-PCR using a CFX 96 Touch Real time PCR Detection System (Bio-Rad).

### 4.7. Preparation of Cell Lysates and Western Blotting Analysis

After 24 h of treatment of CMT-U229 and P114 cells with or without teglicar, cells were harvested, washed with ice-cold PBS, and lysed with ice-cold RIPA buffer in the presence of a protease inhibitor mixture and a phosphatase inhibitor cocktail. Cell lysates were sonicated and then centrifuged at 14,000× *g* for 15 min at 4 °C, and the resulting supernatants were stored at −80 °C until use. Protein concentration was determined using the DC protein assay kit. For western blotting, the cell lysate was mixed with 4× Laemmli sample buffer and boiled for 5 min. Samples (40–60 μg) were loaded into SDS-PAGE and transferred to nitrocellulose membranes. The blot membranes were cut based on standard band positions and then incubated with the corresponding primary antibody overnight at 4 °C with agitation, followed by incubation with an appropriate secondary antibody. Protein bands were visualised using Immobilon Crescendo Western HRP Substrate and then exposed to X-ray film. The antibodies used were rabbit cleaved caspase-3 (1:1000), rabbit cleaved caspase-9 (1:500), rabbit cleaved caspase-8 (1:500), and mouse β-actin (1:20,000). The expression levels of cleaved caspase 3, cleaved caspase-9, and cleaved caspase-8 were quantified, normalised with β-actin from the same blot, and expressed as arbitrary units.

### 4.8. Statistical Analysis

Statistical analysis was performed using GraphPad Prism 9 software (La Jolla, San Diego, CA, USA). All data are presented as the mean ± standard error of the mean (S.E.M.). Comparisons were made using the Student’s *t*-test, one-way analysis of variance (ANOVA), or Two-way ANOVA, followed by a Dunnet post hoc test where appropriate. Results with *p* < 0.05 were considered statistically significant.

## Figures and Tables

**Table 1:** IC_50_ values of cell lines analysed.

Cell Line	Teglicar (μM) IC_50_ ± SEM
P114	7.9 ± 1.280
CMT-U229	13.5 ± 1.483
MDCK	90.9 ± 7.096

IC_50_ values were determined by incubating cell lines with serial dilutions of teglicar for 24 h and determining viability using the Crystal Violet Assay (Merk Life Science, Milan, Italy). The values given are mean ± SEM of three independent determinations.

**Table 2:** List of primers used in this paper.

Gene	Primers
*Rps-5*	Fw: 5′-TCACTGGTGAGAACCCCCT-3′ Rv: 5′-CCTGATTCACACGGCGTAG-3′
*Caspase-3*	Fw: 5′-GCAAACCTCAGGGAAACATT-3′ Rv: 5′-CTTAGAAGCACGCAAACAAAAC-3′
*Caspase-8*	Fw: 5′-GATGCAGATGCGTTGAGTAA-3′ Rv: 5′-AGCCATAGATGATGCCCTTGT-3′
*Caspase-9*	Fw: 5′-GGGAAGCCCAACGTCTTCTT-3′ Rv: 5′-AGTGGGCAAACTAGACACGG-3′

## Data Availability

The data presented in this study are available on request from the corresponding author.

## Supplementary Materials

The following supporting information can be downloaded at: https://www.mdpi.com/article/10.3390/ph16070987/s1; Figure S1: Original blots for CMT-U229 cells; Figure S2: Original blots for P114 cells.
