# Supplementary material for: The Carnitine Palmitoyltransferase 1A Inhibitor Teglicar Shows Promising Antitumour Activity against Canine Mammary Cancer Cells by Inducing Apoptosis
Source: Pharmaceuticals (Basel). 2023 Jul 10;16(7):987. doi: 10.3390/ph16070987 (PMC10383333; doi:10.3390/ph16070987)
Supplement: Supplementary file 1 [file pharmaceuticals-16-00987-s001.zip › pharmaceuticals-2410280-supplementary.pdf]

Full original blots used for Figure 6. Each blot membrane was cut based on the standard band positions and then incubated with the corresponding antibodies.

CMT-U229 cells

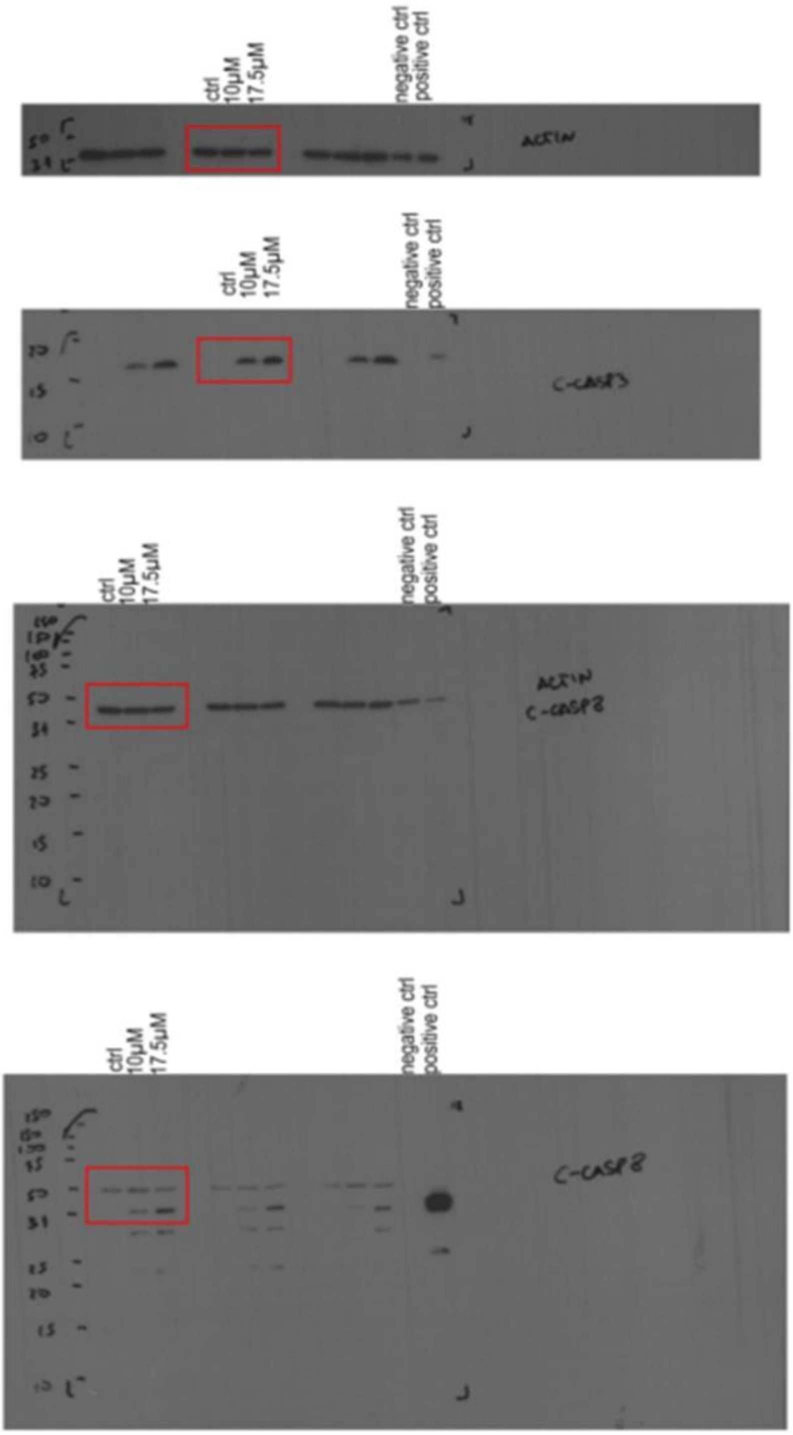

Figure S1: Original blots for CMT-U229 cells

Full original blots used for Figure 6. Each blot membrane was cut based on the standard band positions and then incubated with the corresponding antibodies.

P114 cells

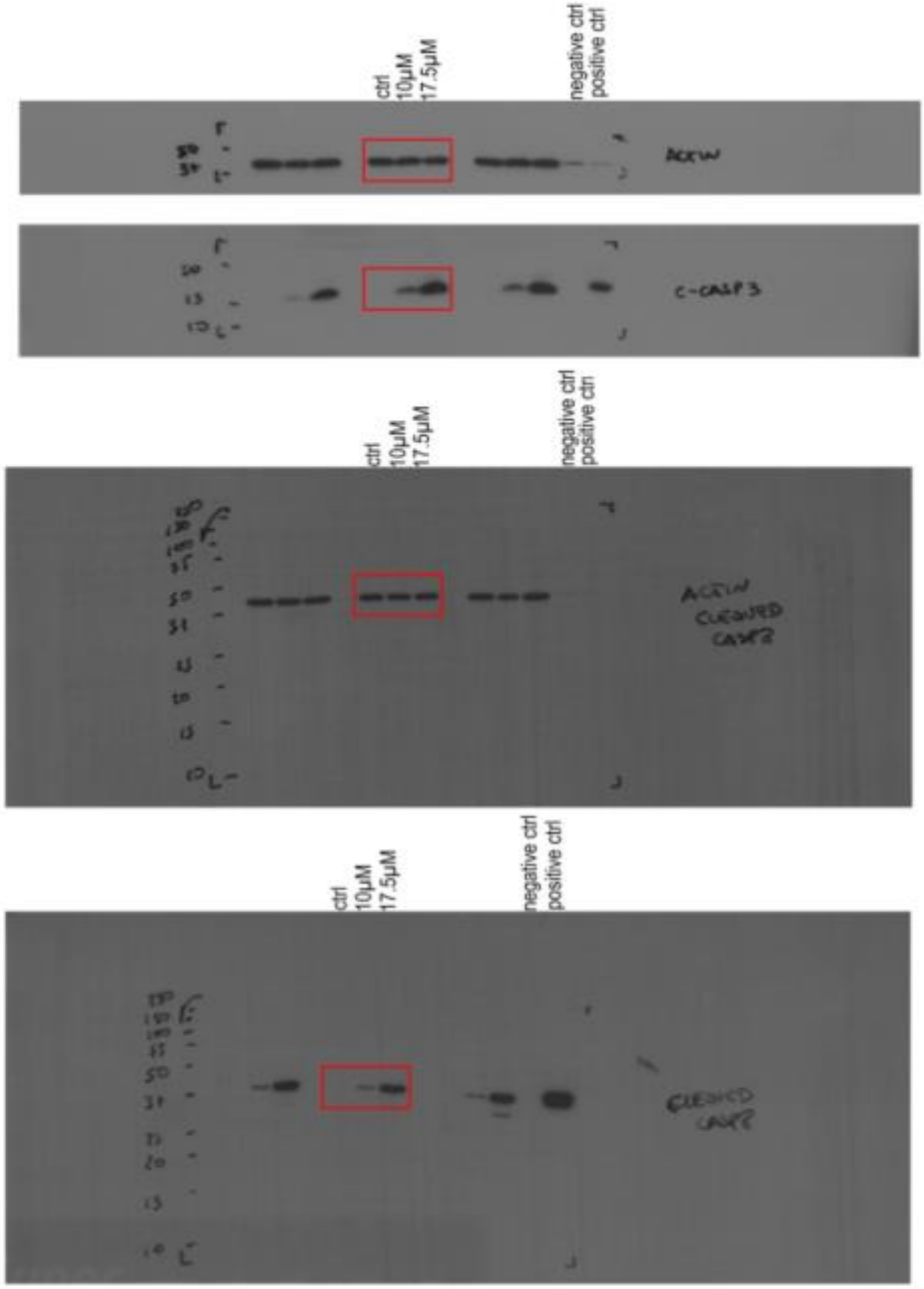

Figure S2: Original blots for P114 cells.
